# Nanostructured Lipid Carriers Loaded with *Lippia sidoides* Essential Oil as a Strategy to Combat the Multidrug-Resistant *Candida auris*

**DOI:** 10.3390/pharmaceutics14010180

**Published:** 2022-01-13

**Authors:** Iara Baldim, Mario H. Paziani, Patrícia H. Grizante Barião, Marcia R. von Zeska Kress, Wanderley P. Oliveira

**Affiliations:** Faculty of Pharmaceutical Sciences of Ribeirão Preto, University of São Paulo, Ribeirão Preto 14025-710, Brazil; iara.baldim@usp.br (I.B.); mariopaziani@alumni.usp.br (M.H.P.); phelena@usp.br (P.H.G.B.); kress@fcfrp.usp.br (M.R.v.Z.K.)

**Keywords:** essential oil, lipid nanoparticles, *Lippia sidoides*, *Candida auris*, COVID-19, antifungal activity, in vivo toxicity

## Abstract

The emerging pathogen *Candida auris* is an emerging fungal pathogen that was associated with nosocomial infectious outbreaks. Its worldwide incidence and the emerging multidrug-resistant strains highlight the urgency for novel and effective antifungal treatment strategies. *Lippia sidoides* essential oil (LSEO) proved antifungal activity, including anti-Candida. However, it may undergo irreversible changes when in contact with external agents without adequate protection. Herein, we encapsulated LSEO in nanostructured lipid carriers (NLC) through the hot emulsification method followed by sonication. NLC matrix was based on oleic acid and Compritol^®^ 888, or a combination of carnauba wax and beeswax, stabilized by sodium dodecyl sulfate. Eight formulations were produced and characterized by the determination of the particle size (213.1 to 445.5 nm), polydispersity index (around 0.3), and ζ-potential (−93.1 to −63.8 mV). The antifungal activity of nanoparticles and LSEO against *C. auris* and the in vivo toxicity in *Galleria mellonella* model were also evaluated. Both NLC and LSEO exhibited potent activity against the yeast, with Minimum Inhibitory Concentration between 281 and 563 µg/mL, and did not evidence toxicity in the in vivo model. Therefore, this study confirms the viability of NLCs loaded with LSEO in combating drug-resistant pathogens as a potential new therapeutic strategy for managing of candidemia.

## 1. Introduction

Since 2009, when it was first isolated in Japan [1], *Candida auris* became a major concern to medical mycology, emerging across the globe in more than 40 countries, being easily transmissible, and associated with high mortality rates [2,3,4]. In addition to being widespread as a multiple drug-resistant pathogen, several other attributes make this fungus a major matter of concern, including the easy spreading in healthcare facilities and the ability to persist for days to weeks both in the human host and on inanimate surfaces [5,6,7]. Moreover, *C. auris* diagnostics in resource-limited countries is another challenge, as it involves complex and expensive equipment, such as MALDI-TOF MS [4]. In addition, of particular concern is that the COVID-19 pandemic overwhelmed the healthcare facilities and provides the ideal conditions for new outbreaks of *C. auris* infection, causing an increase in the patients’ mortality rates [8,9].

The treatment of multiple drug-resistant fungal infections is currently a difficult task due to the limited therapeutic options on the market. *C. auris* presents an even more challenging treatment profile, as it can develop different resistance mechanisms, such as drug target mutation and overexpression, activation of stress response pathways, biofilm formation, and alterations in drug efflux and uptake [10]. The mechanism of biofilm formation plays a key role in *C. auris* antibiotic resistance and propagation in nosocomial environments [11,12]. To hamper this scenario, the production of novel antibiotics and antifungals decreased significantly in the last decades [13]. Hence, the search for new ways to combat the drug-resistant *C. auris* and other human pathogens is extremely needed, being a fascinating research subject. 

Currently, there were reported a significant number of studies aiming to use herbal products (e.g., crude, semipurified and purified plant extracts and essential oils) to fight the multiple drug-resistance of human pathogens [13,14,15,16]. Possible mechanisms of action of herbal products on drug-resistant *Candida* sp., including *Candida* sp., are well described in Herman and Herman [1]. They involve inhibiting the transformation of yeast into hyphae, biofilm formation, reduction of the cytoplasmic membrane biosynthesis or cell wall, production of reactive oxygen species (ROS), and overexpression of membrane transporters. Due to the complex composition of herbal medicines and the synergism between their constituents, these different mechanisms can occur simultaneously. Herbal medicines can be used alone or combined with conventional antimicrobials, opening new perspectives for facing this serious public health problem.

Lipid nanoparticles are biocompatible encapsulation systems attracting widespread interest due to the possibility of expanding the range of molecules to be clinically used, with limited toxicological risk [17]. The encapsulation of EOs in lipid nanoparticles is a strategy to modulate their release properties and enhance their stability and antimicrobial activity [18,19]. Undoubtedly, this technology has the potential to combat multidrug-resistant antimicrobial strains, including *C. auris*. 

An overview in the current literature shows several studies concerning the EOs encapsulation in nanostructured lipid carrier (NLC). Saporito et al. [20] investigated the antimicrobial activity and wound healing properties of lipid nanoparticles loaded by eucalyptus EO. The nanoparticles exhibited good antimicrobial properties against *Staphylococcus aureus* and *Streptococcus pyogenes*, and wound healing properties toward fibroblasts. Baldim et al. [21] encapsulated *Lippia sidoides* EO into lipid nanoparticles and obtained a promising antimicrobial system, with potent activity against *Candida albicans*.

The potent antimicrobial properties of *L. sidoides* EO and its major constituent, thymol, are well described in the literature [21,22,23,24,25]. *L. sidoides* is a Brazilian aromatic plant, popularly known as pepper-rosemary, and commonly used in the traditional medicine. Its leaves are rich in essential oil, whose main bioactives are monoterpenes like thymol, carvacrol, and sesquiterpenes, as caryophyllene [26]. In this paper, we propose to use the NLC as a delivery system to encapsulate *L. sidoides* EO and evaluate its potential as an antimicrobial agent in combating the *C. auris*’ multidrug resistant pathogen. Optimized formulations were produced and characterized by determining the particle size, polydispersity index, and ζ-potential, as well as evaluating their antifungal activity against the multidrug-resistant *C. auris* and the in vivo toxicity using *Galleria mellonella* larvae model. We believe that the technological platform proposed here, and the results obtained, are of great relevance and open perspectives for future proposals for novel EOs’ based antifungal. At the best of our knowledge, such products are not commercially available yet.

## 2. Materials and Methods

### 2.1. Material

Thymol, sodium dodecyl sulfate, and acetonitrile HPLC grade were purchased from Sigma–Aldrich (St. Louis, MO, USA). *L. sidoides* EO, distilled from fresh leaves, was purchased from Pronat (Produtos Naturais LTDA, Horizonte, Brazil). Methanol and oleic acid were purchased from Labsynth (Vinhedo, Brazil). Compritol^®^ 888 ATO (Gattefossé, Saint-Priest, France—melting range from 65 °C to 77 °C), Carnauba wax (Foncepi, Fortaleza, Brazil—melting range from 80 °C to 86 °C), and Beeswax (Via Farma, São Paulo, Brazil—melting range from 61 °C to 65 °C) were the solid lipids used. The NLCs constituents—solid lipids: Compritol^®^ 888 ATO, Carnauba wax and Beeswax; liquid lipid: oleic acid; and surfactant: sodium dodecyl sulfate—are generally recognized as safe substances (GRAS/FDA listed) for use in various pharmaceutical, cosmetics and food products at concentrations used in this work [27,28,29]. More detailed and updated information regarding these substances, including CAS registry number, main physicochemical properties, applications, safety, regulatory status, among others, are presented in Sheskey et al. [29].

### 2.2. Lippia sidoides Essential Oil Characterisation by GC-MS

The main constituents of *L. sidoides* EO were identified by gas chromatography coupled to mass spectroscopy (GC-MS), using the methodology described by Baldim and coworkers (2019) [21]. The EO was previously diluted in methanol at a final concentration of 0.5 mg/mL, and 1 µL of the resulting solution was injected in the gas chromatograph [30]. 

### 2.3. Preparation of the Nanostructured Lipid Carrier Systems

The nanostructured lipid carrier systems loaded with *L. sidoides* EO were prepared by hot emulsification using high-speed homogenization followed by ultrasonication (US), according to the procedure previously reported by Baldim and coworkers [21]. The solid lipid was melted at 10 °C above its melting point and mixed with the oleic acid (liquid lipid) and the *L. sidoides* EO. The aqueous phase was constituted by sodium dodecyl sulfate (SDS) dissolved in a 10 mM pH 7.0 phosphate buffer saline (PBS) at different ratios. Table 1 presents the resulting NLC systems. The aqueous phase was heated and gently dispersed into the lipid phase and homogenized for 3 min at 14,000 rpm/min (UltraTurrax T18, IKA-Works, Wilmington, NC, USA). The hot oil-in-water nanostructured lipid system was further submitted to ultrasonication in an ultrasonic sonicator VCX-750 (SONICS Vibracell, Newtown, CT, USA), equipped with a 13 mm diameter probe. The US intensity was fixed at 45% of amplitude, at frequency of 20 kHz, applied for 5 cycles of 2 min ON and 1 min OFF. The samples were maintained at a constant temperature of 85 °C in a water bath.

### 2.4. Droplet Size, Polydispersity Index, and ζ-Potential

The dynamic light scattering was used to measure the size and the polydispersity index of the EO-loaded NLCs. The measurements were made in a Zetasizer Nano—ZS90 (Malvern, UK). The samples were diluted 1:200 using a 10 mM PBS at pH 7.0, to minimize multiple scattering effects. In addition to the mean hydrodynamic radius (z-average) of the particles, the equipment also reports the polydispersity index (PI), which ranges from 0 (monodisperse) to 1 (very broad distribution). ζ-potential was also determined by micro electrophoresis in the same instrument. The measurements were carried out in triplicate at 25 °C.

### 2.5. Thymol Retention of Liquid Emulsions

Thymol, the major component present in *L. sidoides* EO was used as marker compound to evaluate the encapsulation process. The high-performance liquid chromatography coupled to a diode arrangement detector (HPLC-DAD) was used to quantify the thymol present in the EO-loaded NLCs systems. The chromatographic method and conditions used followed the method proposed by Leal and coworkers (2003) [31]. 

### 2.6. Antifungal Activity

The antifungal profile of the EO-loaded NLCs and *L. sidoides* EO against the multidrug-resistant *C. auris* (CDC B11903) was performed both by a previous screening analysis to determine the sensitivity, and by determining the minimum inhibitory concentrations (MIC) and minimum fungicidal concentration (MFC).

The screening for anti-*Candida* activity was performed by agar diffusion assay to evaluate the growth inhibition of *C. auris* by EOs and EO-loaded NLC formulations. The inoculum was prepared using 24-h plate cultures of *C. auris* in Sabouraud Agar dextrose incubated at 37 °C. The colonies were suspended in 0.85% saline buffer in suspension of 1 × 10^6^ cells/mL. The RPMI1640 agar supplemented with glucose 2.0 g/L was inoculated with the *C. auris* suspension. A drop of 10 µL of each NLC formulations and 5 µL of the pure EO were placed on the top surface of the inoculated agar plate, which was incubated at 37 °C for 24 h. The results were evaluated by the presence or absence of inhibition zones.

Antifungal susceptibility testing was performed in accordance Clinical and Laboratory Standards Institute (CLSI) guidelines for broth microdilution test (protocol M27-A3) [32] against the multidrug-resistant *C. auris*, with modifications. As negative controls, it was prepared blank formulations based on the solid lipid Compritol ATO (F8B), and one for compositions based on the solid lipid B+CW (beeswax plus carnauba wax 1:1 ratio-F17B), at the intermediary concentrations of the anionic surfactant used (2.8% of sodium dodecyl sulphate-SDS). A control of pure SDS at a concentration of 2.8% was also used in the antifungal assay. The final EO and EO-loaded NLC formulations concentration in the plates ranged from 2.250 to 4.4 × 10^−3^ mg/mL. The yeast inoculum was prepared in a final concentration of 4 × 10^5^ CFU/mL and inoculated in RPMI-1640 (Life Technologies, Grand Island, NY, USA) buffered to pH 7.0 with MOPS (USB Corporation, Cleveland, OH, USA) and supplemented with L-glutamine (0.3 g/L) and D-glucose (2.0 g/L). The plates were incubated at 37 °C and readings were taken at 24 h and 48 h of fungus development. The minimum inhibitory concentration (MIC) was considered as the lowest concentration able to inhibit fungal growth. For analysis of the results, geometric means (GM) and MICs’ ranges were calculated. The minimum fungicidal concentration (MFC) of the EO and EO-loaded NLCs was evaluated by the inoculation of 10 µL of MIC well on Sabouraud Dextrose Agar (SDA) media culture (Acumedia Neogen^®^, Lansing, MI, USA). The plates were incubated at 37 °C for 5 days and it is evaluated the presence or absence of fungus growth. All experiments were carried out in duplicate. 

### 2.7. In Vivo Toxicity Assay

The toxicity of EO-loaded NLCs was determined in a *Galleria mellonella* in vivo model [33]. *G. mellonella* is a well acceptable model to study toxicity levels of new compounds, drug treatments, and also a systemic infection model to study different pathogens, including *Candida albicans* and *Candida auris* [34,35]. Here, we randomly divided groups of 10 *G. mellonella* larvae and individually weighed prior to treatment (larvae weighing between 250−350 mg each were used). The tested concentrations were based on the MIC concentrations: sub-inhibitory (low), minimum inhibitory concentration (MIC) and supra inhibitory (high) (data presented on Table 2). The toxicity profile of pure EO and at a 1:2 (*v*/*v*) dilution was also determined. The artificial inoculation was performed by injecting 5 μL of each NLC in each concentration (low, MIC and high), using a Hamilton micro syringe for gas chromatography model 7000.5 KH, 10 μL. Doses were injected into the last right proleg of the larvae. Naïve larvae (a group of larvae without intervention) and phosphate buffered saline (PBS) was used as a negative control for toxicity. Control groups of larvae received 10 µL of sterile PBS in the same manner. Between injections, the micro syringe was rigorously washed 3 times with sodium hypochlorite, 70% ethanol, and autoclaved water. Inoculated larvae were deprived of feed, incubated at 37 °C and scored for viability at 24-h intervals. Differences in resulting survival plots were evaluated using the Mantel–Cox test (Log-rank method) using the GraphPad Prism 5 software. The EO-loaded NLC formulations, at their respective concentrations tested, were considered toxic when they were able to kill at least 50% of larvae in a period of 5 days postinjection, following the protocol of Gottardo et al. (2019) [36].

### 2.8. Statistical Analysis

The statistical significance of the experimental data was determined by one-way analysis of Variance (ANOVA). Significant differences of means were determined using Tukey’s multiple comparison test with a statistical significance of *p* ≤ 0.05. 

## 3. Results and Discussion

### 3.1. Chemical Characterization of Lippia sidoides Essential Oil

The GC-MS analysis allowed the identification of twenty-six components in the LSEO (Table 3). Thymol, the bioactive molecule associated to the biological properties of this EO [37], was the major constituent detected (68.22%) followed by p-cymene (9.43%), trans-caryophyllene (7.72%), β-myrcene (2.84%), γ-terpinene (2.71%), and α-terpinene (1.16%). These six constituents represent 92.08% of the total detection area.

### 3.2. Characterization of the NLCs

The polydispersity index (PI) is a parameter to determine the homogeneity of particle sizes and their dispersion quality. PI values range from 0 to 1, and the lower the value, the better the colloidal suspension quality [38]. ζ-potential characterizes the surface charge of the NLC particles with information concerning the repulsion forces between the particles. Values above 30 mV (in module), favor the repulsion between the particles, prevent coalescence and increase the system stability [39]. The results of the NLCs’ characterization are shown in Table 4.

The composition of the NLCs affected both particle size and PI. NLC composed of beeswax and carnauba wax presented smaller (301.2 to 318.7 nm) and more homogeneous (0.18 to 0.22) particles compared to those composed of Compritol^®^ 888 (whose sizes varied between 213.1 and 445.5 and the PI between 0.25 and 0.41). The effect of surfactant concentration was significantly more pronounced in formulations composed of Compritol^®^ 888 as solid lipid: higher concentration of SDS provided smaller and less polydisperse particles.

SDS is an anionic surfactant, employed in this study to stabilize different hydrophobic matrices: a pure Compritol^®^ 888 matrix and a mixture (1:1) of beeswax and carnauba wax. Surfactants are characterized by parameters such as CMC (critical micelle concentration) and HLB (hydrophilic-lipophilic balance) [40]. CMC is defined as the concentration of surfactants when the micelles spontaneously form, while HLB is a parameter to qualify the surfactant’s emulsification properties. Both of these definitions are closely related [41]. The micelle formation process depends on two factors: the electrostatic interactions between the charged head groups of the components; and the hydrophobic interactions between the hydrocarbon tails of components [42]. The more hydrophobic the chain, the lower the CMC, so micelles are formed at a lower surfactant concentration. Upon reaching CMC, any further addition of surfactants will only increase the number of micelles, not interfering with particle size. Compritol^®^ 888 ATO has an amphiphilic character, due to acylglycerols in the composition [43]. On the other hand, the mixture of beeswax and carnauba wax gives strong lipophilic properties to the nanoparticles and, therefore, contributes to reducing the CMC, which justifies the fact that the concentration of SDS did not influence the particle size of these formulations.

The nanoparticle charge is one of the factors related to the physical stability of the system. All formulations presented negative ζ-potential values, in the range −63.8 to −93.1 mV, and this parameter was significantly affected by the composition of the formulations. High negative or positive electrical charge avoids the occurrence of aggregation, once the charges tend to repel each other [44]. For this study, NLC containing a combination of beeswax and carnauba wax had significantly higher values of ζ-potential (from −87.5 to −93.1 mV), which indicates greater electrostatic stability in comparison to nanoparticles composed of Compritol^®^ 888 ATO as solid lipid (from −63.8 to −77.0 mV). The negative charge is related to the presence of negatively charged head groups of the anionic surfactant SDS exposed in the outer region of the nanoparticles [45].

**Figure 1:**
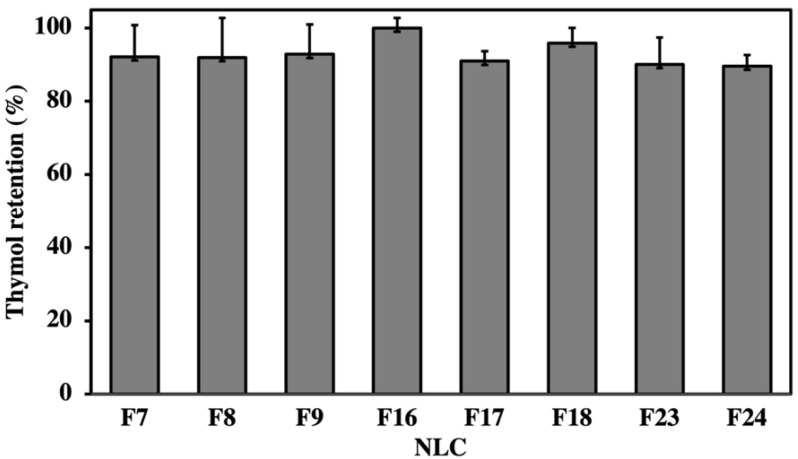
Thymol retention of NLC loaded by *Lippia sidoides* essential oil, quantified by HPLC-DAD.

All EO-loaded NLC formulations retained a high percentage of the marker compound thymol, the major constituent of *L. sidoides* EO (Figure 1). The retention values varied from 90% to 100% and did not change significantly by increasing the concentration of surfactant or modifying the lipid matrix’s composition (*p* < 0.05). Some factors are closely related to a high loading capacity of NLCs, such as the solubility of the encapsulated material in the lipid matrix, type and concentration of the surfactant, type of lipid matrix and the ratio of solid and liquid lipids forming the core of NLC [17]. Yue et al. successfully loaded bupivacaine (BPV) into NLCs and reported very high encapsulation efficiency, i.e., 90% [46]. Baldim et al. developed a lipid nanosystem to encapsulate *L. sidoides* EO and obtained fairly high rates of thymol retention, in the range of 91–100% [21]. In our study, the high levels of thymol retention can be attributed to the lipophilic nature of the essential oil and NLCs that subsequently causes higher thymol partitioning into lipid matrix of NLC and lesser into the aqueous phase [47].

### 3.3. Antifungal Activity against Multidrug-Resistant Candida auris

**Figure 2:**
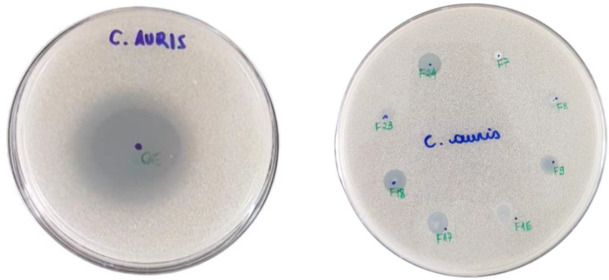
Agar diffusion test of *L. sidoides* essential oil (**left**) (5 µL) and EO-loaded NLC formulations (**right**) (10 µL) against CDC B11903 strain of multidrug-resistant *C. auris*. A drop of 10 µL of each NLC formulation and 5 µL of the pure EO were placed on top surface of agar plate, and antifungal activity was evaluated by presence or absence of inhibition zones. NLC compositions are described in Table 1.

The in vitro antifungal activity of *L. sidoides* EO-loaded NLCs against multidrug-resistant *C. auris* was first assayed by agar diffusion test. EO and EO-loaded NLC formulations showed an inhibition zone. F9, F16, F17, F18, and F24 EO-loaded NLCs formulations exhibited larger inhibition zones (Figure 2).

The broth microdilution assays allowed the determination of the MICs of *L. sidoides* EO and EO-loaded NLCs against *C. auris*. Table 2 summarizes the MIC and MFC for the EO, EO-loaded NLC, and NLC without essential oil against *C. auris*. As a quality control for the experiment, we included a negative control with saline and RPMI to ensure the purity of both, without yeast and bacterial contamination. Both showed *C. auris* growth in all the analyzed dilutions. Regarding the tested samples, the highest anti-*Candida* activity was achieved with EO and EO-loaded NLCs containing higher SDS concentrations in the composition (F8, F9, F17, F18 and F24), which exhibited the lowest MIC values. *Candida auris* was inhibited by both EO and EO-loaded NLC with concentrations between 0.281 and 0.563 mg/mL. *L. sidoides* EO and its major isolated compound (thymol) have already been shown to be highly effective in ATCC strains of *C. albicans* [21]. However, in this previous study, both EO and thymol showed lower MIC against yeast than the EO-loaded lipid nanoparticles. Herein, F8, F9, F17, F18, and F24 formulations exhibited similar MIC values, close to that showed by the EO, exhibiting the highest antifungal activities against *C. auris* in the tested samples. In addition, all EO-loaded NLC showed a fungicidal profile in the minimal inhibitory concentrations (Table 2). Despite having different combinations of solid lipids, these formulations contain the highest concentration of SDS in the composition. SDS was active with a MIC value close to that of pure EO (0.281 mg/mL, in EO equivalent, a normalization made for samples without EO representing the amount of EO that would have in that amount of component).

Although obtained synthetically, SDS possess the GRAS status (Generally Recognized as Safe), and is widely used in cosmetics and oral and topical pharmaceutical formulations [29]. Therefore, the NLC can be added as an active ingredient in pharmaceutical formulations up to a 10 mg/mL concentration, being highly effective against *C. auris* and having a final SDS concentration of less than 1%, considering the products obtained at intermediary surfactant concentration.

The mechanism of antifungal action of *L. sidoides* EO is related to its functional groups. The main class of bioactives in the *L. sidoides* EO are terpenes, class of volatile compounds formed by a variety of functional groups [48]. Although there is a limited understanding of the mechanisms of antifungal resistance of *C. auris*, several gene families encoding proteins associated with mechanisms of resistance were identified [49]. Additionally, it was already shown that the complex composition and the combination of EO constituents present synergistic effects, simultaneously attacking different targets of a microorganism cell [50,51]. On the other hand, the influence of anionic surfactants (as SDS) on enzyme activity through binding to enzymatic proteins was extensively demonstrated [52]. Furthermore, the exposure of *C. auris* cells to SDS is related to the denaturation of cell wall proteins and lipid damage [6]. The SDS positively affects antifungal activity, which may be related to the interaction of the anionic surfactant molecules with proteins and lipid content of the cell wall. Based on these observations, we hypothesized that the synergy between the different EO constituents, which presents different functional groups, favored the high antifungal activity of the samples. In addition, the higher the concentration of anionic surfactant in the samples, the greater the availability for the EO constituents to interact with the yeast cell wall, which explains the same MIC values for the free EO and the formulations F8, F9, F17, F18, and F24.

Despite the need for deeper studies about the mechanism of action, the interactions between nanoparticles and the fungal cell wall, and the modulation of *L. sidoides* EO release, it is undeniable that the MIC values obtained (between 0.281 and 0.563 mg/mL) evidenced that the NLCs here developed are highly effective systems to fight the multidrug-resistant *C. auris*.

### 3.4. In Vivo Toxicity

**Figure 3:**
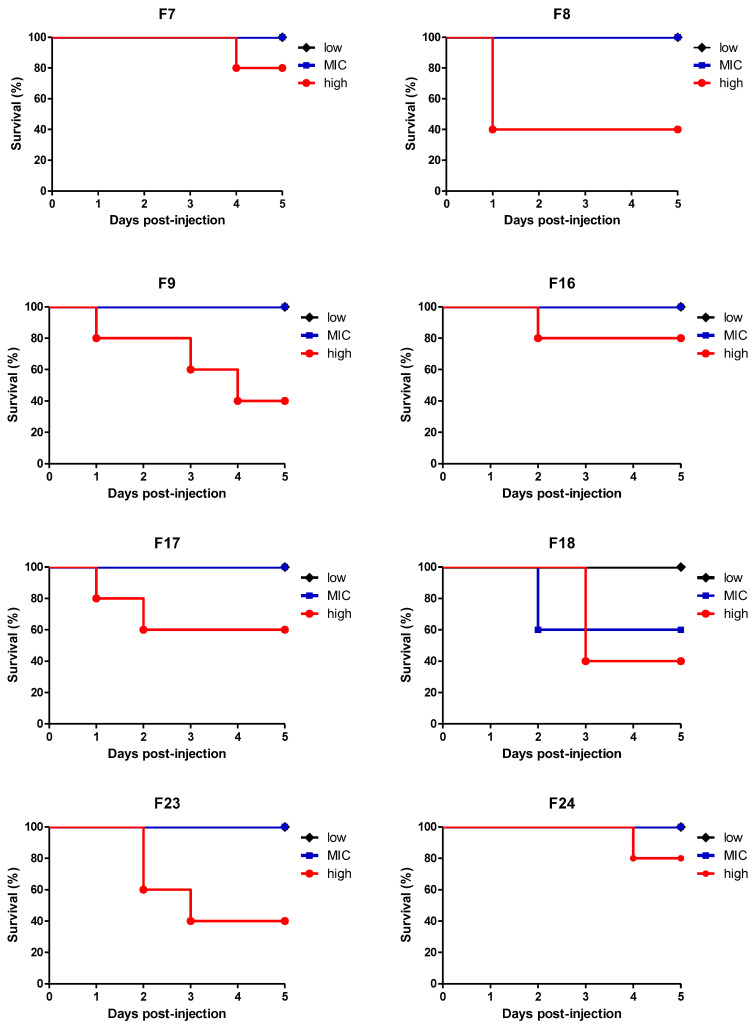
In vivo toxicity assay of *L. sidoides* OE-loaded NLC formulations in *G. mellonella* larvae. Plots of *G. mellonella* survival after injection of 5 μL of NLC at different concentrations: low (dilution below MIC), MIC (at minimal inhibitory concentration), and high (dilution above the MIC). Samples were considered toxic when they could kill at least 50% of larvae within 5 days postinjection. No larval killing was observed in *naïve* and control larvae injected with an equivalent volume of PBS.

The in vivo toxicity assays of the EO-loaded NLCs were performed by the method of injection in *Galleria mellonella* larvae. A peculiarity of this model is its similarity with the immune and toxic responses of mammals. The larval hematocytes act as phagocytes and release proteins with close similarity to mammalian antibodies [36,53,54]. *L. sidoides* EO and EO-loaded NLC formulations were tested with the MIC concentrations against *C. auris* and additionally one concentration below (low) and one above (high) the MIC (Figure 3).

All but one EO-loaded NLC formulation with the concentration below (low) and MIC, did not kill the larvae. F18 EO-loaded NLC killed 60% of the larvae. The EO-loaded NLC formulations containing lower and intermediate concentrations of SDS in the composition (F7, F16, F17, and F24) and with the concentration above the MIC (high), also did not kill the larvae, which demonstrates that the toxicity profile may be related to high anionic surfactant content in the nanoparticles. Regarding the *L. sidoides* EO, the 1:2 (*v*/*v*) dilution killed 40% of the larvae, whereas the pure *L. sidoides* EO killed all the larvae 24 h postinjection. The negative toxicity control (PBS), as expected, was not able to kill any larvae, presenting a non-toxic profile (data not shown). Moreover, these findings reinforce the excellent performance of NLCs as highly biocompatible carrier systems, which are generally assumed as safe constituents of several pharmaceutical, cosmetics and food products [27,28,29].

## 4. Conclusions

We showed that *L. sidoides* EO-loaded NLCs can be successfully produced by the hot emulsification method followed by ultra-sonication. Different characterization’s methods confirmed the formation of particles in the nanometric range, with high retention efficiency of *L. sidoides* EO. Regarding the composition of nanoparticles, the concentration of SDS was a key factor to the product properties: NLC containing high SDS content presented smaller and less polydisperse nanoparticles, with good thymol retention and strong activity against *C. auris*, comparable to *L. sidoides* EO antifungal activity. The pure SDS exhibited an antifungal activity similar to that of the pure *L. sidoides* EO, but the blank formulations tested (F8B and F17B) did not show antifungal activity, at the concentrations range assayed.

Furthermore, the in vivo nontoxic profile of EO-loaded NLC formulations observed for *G. mellonella* larvae suggest a close relationship with the enhanced biocompatibility of the lipid matrix. Finally, the high performance in anti-*C. auris* assays and low toxicity of EO-loaded NLC formulations highlight this study’s high relevance and novelty. To the best of our knowledge, this is one of the few successful strategies to effectively fight *C. auris*, and it opens perspectives for further studies with EO and EO-loaded NLCs as an effective approach to combat multidrug-resistant pathogens.

## Figures and Tables

**Table 1:** *Lippia sidoides* EO-containing NLC prepared with variable surfactant and lipid content. Quantities are expressed as % *w/w*.

Form.	Surfactant	Lipid Matrix			EO	10 mM pH 7.0 PBS (q.s.)
		Solid Lipid		Liquid Lipid		
	SDS	Compritol^®^ 888 ATO	BW + CW	Oleic Acid		
F7	1.4	5.2	0	1.8	1.8	100
F8	2.8	5.2	0	1.8	1.8	100
F9	4.2	5.2	0	1.8	1.8	100
F16	1.4	0	5.2	1.8	1.8	100
F17	2.8	0	5.2	1.8	1.8	100
F18	4.2	0	5.2	1.8	1.8	100
F23	2.8	5.2	0	1.8	1.8	100
F24	2.8	0	5.2	1.8	1.8	100

BW: beeswax; CW: carnauba wax; EO: *L. sidoides* essential oil.

**Table 2:** Minimum inhibitory concentration (MIC) and minimal fungicidal concentration (MFC) of *L. sidoides* EO, EO-loaded NLC, and NLC without essential oil against CDC B11903 strain of multidrug-resistant *C. auris.*

NLC	Geometric Mean MIC (mg/mL)	MIC Range (mg/mL)	MFC (mg/mL)
*L. sidoides* EO	0.281	0.563–0.140	0.563–0.140
F7	0.563	1.125–0.281	1.125–0.281
F8	0.281	0.563–0.140	0.563–0.140
F9	0.281	0.563–0.140	0.563–0.140
F16	0.563	1.125–0.281	1.125–0.281
F17	0.281	0.563–0.140	0.563–0.140
F18	0.281	0.563–0.140	0.563–0.140
F23	0.563	1.125–0.281	1.125–0.281
F24	0.281	0.563–0.140	0.563–0.140
F8B *^,1,2^	>2.250	>2.250	>2.250
F17B *^,1,2^	>2.250	>2.250	>2.250
SDS *	0.281	0.281	0.281

* EO equivalent in the formulations at intermediary SDS concentration (F8 and F17) ^1^: Fungal Growth at all concentrations assayed ^2^: NLC without EO.

**Table 3:** Chemical constituents of *Lippia sidoides* EO *.

Compound	% ^a^	Kovats Index ^b^
α-Thujene	0.95	928
Bicyclo[3.1.1]hept-2-ene, 2,6,6-trimethyl	0.56	937
2-β-Pinene	0.16	980
β-Myrcene	2.84	989
1-Phellandrene	0.07	1007
E-β-Ocimene	0.16	1011
α-Terpinene	1.16	1018
p-Cymene	9.43	1025
Bornylene	0.64	1032
1,8-Cineole	0.53	1033
1,3,6-Octatriene, 3,7-dimethyl-, (Z)-(CAS)	0.11	1037
1,3,6-Octatriene, 3,7-dimethyl-, (E)-(CAS)	0.16	1048
γ-Terpinene	2.71	1060
Linalyl acetate	0.35	1099
2-(Chloromethyl)tetrahydropyran	0.15	1145
Bicyclo[3.1.0]hex-3-en-2-one, 4-methyl-	0.26	1170
3-Cyclohexen-1-ol, 4-methyl-1-(1-methylethyl)	0.68	1180
Thymol methyl ether	0.97	1231
Thymol	68.22	1297
α-Copaene	0.34	1377
trans-Caryophyllene	7.72	1420
Aromadendrene	0.46	1440
α-Caryophyllene	0.34	1455
Ledene	0.44	1493
δ-cadinene	0.10	1520
Caryophyllene oxide	0.43	1580

^a^ Percentages were calculated based on normalized MS peak areas. ^b^ Kovats Index: retention index relative to a series of alkanes (C10–C22) * Reprinted with permission from Baldim et al. [21]. Copyright 2019. Elsevier—License number 5213111032701.

**Table 4:** Characterization of NLCs loaded by *Lippia sidoides* essential oil.

NLC	Size (nm)	PI (-)	ζ-Potential (mV)
F7	445.5 ± 8.7 ^a^	0.41 ± 0.01 ^a^	−63.8 ± 8.7 ^a^
F8	328.0 ± 8.7 ^b^	0.33 ± 0.04 ^b^	−74.0 ± 10.7 ^b,c^
F9	213.1 ± 1.7 ^f^	0.25 ± 0.01 ^c,d^	−72.0 ± 8.2 ^b^
F16	318.7 ± 2.3 ^c^	0.20 ± 0.00 ^d^	−87.5 ± 2.8 ^d^
F17	307.8 ± 3.0 ^d,e^	0.22 ± 0.03 ^d^	−93.1 ± 2.7 ^d^
F18	301.2 ± 4.8 ^e^	0.18 ± 0.03 ^d^	−90.1 ± 4.2 ^d^
F23	321.3 ± 10.9 ^b,c^	0.33 ± 0.04 ^b^	−77.0 ± 7.4 ^c^
F24	301.5 ± 5.0 ^e^	0.19 ± 0.02 ^d^	−89.5 ± 3.0 ^d^

Same letter means no significant difference according to Tukey´s multiple comparison test (*p* < 0.05).
